# High Rate of Loss to Follow-Up Among Patients Undergoing Treatment for Premalignant Cervical Lesions at Mbarara Regional Referral Hospital, Southwestern Uganda: A Retrospective Cohort Study

**DOI:** 10.7759/cureus.46542

**Published:** 2023-10-05

**Authors:** Rogers Kajabwangu, Frank Ssedyabane, Deusdedit Tusubira, Samuel Maling, Natthan Kakongi, Eleanor Turyakira, Alexcer Namuli, Martin Galiwango, Thomas C Randall

**Affiliations:** 1 Department of Obstetrics and Gynecology, Mbarara University of Science and Technology, Mbarara, UGA; 2 Department of Medical Laboratory Science, Mbarara University of Science and Technology, Mbarara, UGA; 3 Department of Biochemistry, Mbarara University of Science and Technology, Mbarara, UGA; 4 Department of Psychiatry, Mbarara University of Science and Technology, Mbarara, UGA; 5 Department of Community Health, Mbarara University of Science and Technology, Mbarara, UGA; 6 Department of Obstetrics and Gynecology, Mbarara Regional Referral Hospital, Mbarara, UGA; 7 Department of Electrical and Electronics Engineering, Faculty of Applied Sciences and Technology, Mbarara University of Science and Technology, Mbarara, UGA; 8 Department of Obstetrics, Gynecology, and Reproductive Biology, Harvard Medical School, Boston, USA

**Keywords:** southwestern uganda, adherence, follow-up, loss, premalignant cervical lesions

## Abstract

Background: For a cervical cancer control program to be effective in reducing the incidence of the disease, there should be high compliance to treatment and follow-up of women diagnosed with precursor lesions. Screening programs in low-resource countries such as Uganda are challenged by poor adherence to follow-up following treatment for premalignant cervical lesions. This study sought to describe the burden and factors associated with loss to follow-up among women undergoing treatment for premalignant cervical lesions at a tertiary hospital in southwestern Uganda.

Methods: This was a retrospective cohort study. We reviewed the registers at the Mbarara Regional Referral Hospital (MRRH) cervical cancer clinic for a period of four years from January 2017 to December 2020. Data on age, district of residence, diagnosis, date of diagnosis, date and type of initial treatment, and date of follow-up visit were collected. We also captured data on whether patients returned on the scheduled follow-up date or within three months after the scheduled follow-up date. We defined loss to follow-up as failure to return for follow-up either on the scheduled date or within three months after the scheduled date.

Results: Out of the 298 patients who underwent treatment for premalignant cervical lesions in the study period, 227 (76.2%) did not return for follow-up at one year. At bivariate analysis, failure to attend the review visit at six weeks predicted the loss to follow-up at one year following treatment for premalignant lesions almost perfectly (risk ratio (RR)=2.84, 95% confidence interval (CI): 2.18-3.71, p<0.001). Negative HIV serostatus and receiving thermocoagulation slightly increased the risk of getting lost to follow-up, while being more than 45 years old reduced the odds. At multivariate analysis, treatment with thermocoagulation (adjusted risk ratio (aRR)=1.21, 95% CI: 1.07-1.36, p=0.03) was associated with loss to follow-up at one year.

Conclusion: The proportion of women who did not return for follow-up at one year following treatment for premalignant cervical lesions at Mbarara Regional Referral Hospital is very high. There is a need to implement strategies such as telephone-aided reminders to prompt patients to return for follow-up following treatment for premalignant cervical lesions.

## Introduction

Worldwide, cervical cancer is the fourth most common cancer among women [1]. In 2018, there were 570,000 new cases of cervical cancer and 311,000 deaths from the disease [2]. Approximately 84% of all new cervical cancers and 88% of all deaths from the disease occurred in low-income countries [3]. Compared with other cancers, the mean age at diagnosis of cervical cancer is quite low (53 years), generating a proportionally greater loss of life years [3,4]. Preventive measures for cervical cancer were revolutionized by the discovery of the causative relationship of cancer with persistent high-risk human papillomavirus (HPV) infection [5]. The World Health Organization (WHO) has now set a target to eliminate cervical cancer as a public health problem by 2030 [6]. The strategies to meet this target include fully vaccinating 90% of girls with the human papillomavirus (HPV) vaccine by 15 years of age, screening 70% of women with a high-performance test two times per life by 35 and 45 years of age, and ensuring that 90% of women identified with cervical disease receive treatment and care [6]. In working toward improved screening for cervical cancer, the WHO has recommended a single-visit approach where a patient is treated on the same day following a diagnosis of a premalignant lesion [7]. This approach has been adopted by the Uganda Ministry of Health, using mainly visual inspection methods for cervical cancer screening [8]. The screening program in Uganda is however challenged by a number of factors including low staffing and poor infrastructure [9]. As a result, few women are screened, with many of them presenting with late-stage cancer at diagnosis [10].

Patients who screen positive for premalignant lesions are treated with cryotherapy, thermocoagulation, cold knife conization, and loop electrosurgical excision procedure [11]. For a screening program to be effective in terms of reducing cervical cancer incidence, there should be high compliance with treatment and follow-up of the women diagnosed with the precursor lesions [12]. The WHO recommends a follow-up visit at one year following treatment for premalignant cervical lesions to assess for recurrence [13]. However, many screening programs, especially those in low-resource countries such as Uganda, are challenged by patients’ noncompliance to follow-up visits following treatment for premalignant cervical lesions [14-16]. These patients may have undetected recurrence of premalignant lesions, which may progress to invasive cervical cancer [17].

This study sought to describe the challenge of loss to follow-up among women who receive treatment for premalignant lesions at the cervical cancer clinic of Mbarara Regional Referral Hospital (MRRH) and the sociodemographic factors associated with this challenge.

This article was previously posted to the Research Square preprint server on May 10, 2023.

## Materials and methods

Study setting

The study was carried out at the cervical cancer clinic of Mbarara Regional Referral Hospital, a tertiary hospital located in southwestern Uganda with a catchment area of approximately four million people [18]. The clinic runs five days a week, attending to over 250 clients per month. It is run by three nursing staff, one resident, and three gynecologists supervised by a gyne-oncologist. Screening for cervical cancer is done using visual inspection methods with or without colposcopy, conventional cytology, and HPV DNA. Those with premalignant lesions are treated with cryotherapy and thermocoagulation. Following treatment, the patients are advised to return for a review visit at six weeks for assessment of possible complications and a follow-up visit after one year to assess for recurrence of disease.

Study design and sampling procedure

We performed a retrospective records review of the clinic registers of the cervical cancer clinic for a period of four years from January 2017 to December 2020. We purposely selected all patients who had undergone treatment for any cervical lesion and gave appointment dates for the week 6 and one-year follow-up visits.

Data collection

We designed a data collection tool in REDCAP to ease the management of data extracted from the cervical cancer screening registers. After selecting the registers of interest, we identified the patients who had undergone treatment for premalignant lesions. Data on their demographic characteristics and the treatment given were then collected. We also examined whether or not they returned on the scheduled date or within three months after the scheduled date. Loss to follow-up was defined as failure to return for review on the scheduled date or three months thereafter.

Eligibility criteria

We included only those patients who had undergone treatment for premalignant cervical lesions and had been given follow-up dates for review. We excluded all patients with incomplete clinical records.

Data management and analysis

Data were imported from REDCAP into a Microsoft Excel spreadsheet version 15.0.4675.1003 (Microsoft Inc., Redmond, WA) and then imported into STATA 17 (StataCorp LLC, College Station, TX) software for analysis. Demographic data were presented in the form of frequencies and percentages. The proportion of women who were lost to follow-up was presented as a percentage of all the patients who underwent treatment for premalignant cervical lesions using a bar chart. In the bivariate analysis, we used the log-binomial regression analysis with robust standard errors to determine factors independently associated with loss to follow-up and expressed the results using risk ratio (RR) with the respective 95% confidence interval (CI). The RR was preferred over the odds ratio (OR) because our outcome was frequent. Accordingly, the OR would overestimate the degree of association compared to the RR [19,20]. In the multivariate analysis, we considered variables that had a p-value of <0.2 at bivariate analysis. A p-value of <0.05 was considered to be statistically significant. We tested for multicollinearity using a variance inflation factor (VIF) of ≥10.

## Results

Characteristics of the participants

We extracted data from 298 patients who underwent ablative therapy between January 2017 and December 2020. The mean age of the participants was 32.6±7.7years, and the majority of them (94%, 280/298) were below 45 years. The majority of the participants were residents of Mbarara district, where the hospital is located. Most of the women identified, however, resided more than 10 km away from the hospital. The majority (73.8%, 220/298) of the participants were HIV-negative and were treated with cryotherapy (70.1%, 209/298). Most of them did not come for the review visit at six weeks. The rest of the details are shown in Table 1.

**Table 1:** Baseline characteristics of the participants *Continuous variable, median (IQR) IQR: interquartile range

Characteristic	Categories	Frequency (number (%))
Age	Median age (IQR)	32 (13)*
	45 and below	280 (94)
	More than 45	18 (6)
District of residence	Mbarara	163 (54.7)
	Other districts	135 (45.3)
Distance from the hospital	Less than 10 km	88 (29.5)
	10-30 km	105 (35.2)
	30-60 km	19 (6.4)
	>60 km	86 (28.9)
HIV status	Positive	78 (26.2)
	Negative	220 (73.8)
Treatment given	Cryotherapy	209 (70.1)
	Thermocoagulation	89 (29.9)
Attendance of review visit at six weeks	Attended	104 (34.9)
	Did not attend	194 (65.1)

Proportion of participants lost to follow-up at one year

**Figure 1:**
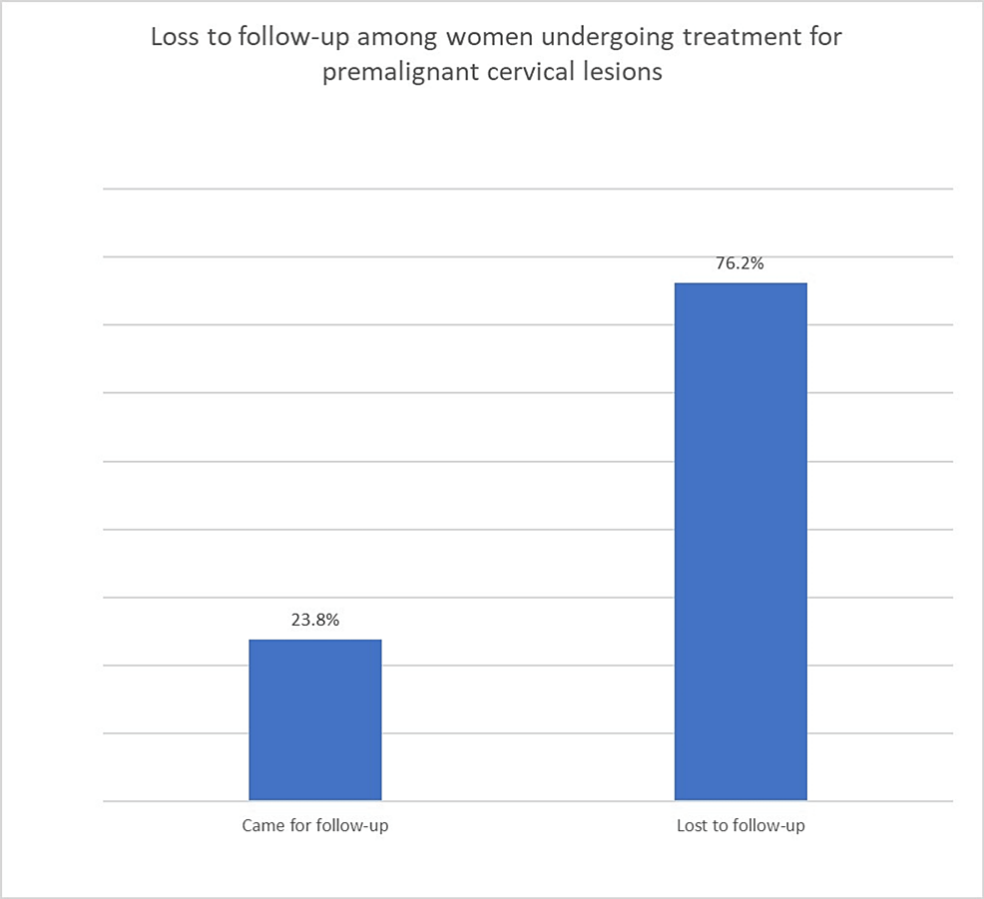
Proportion of women undergoing treatment for premalignant cervical lesions who were lost to follow-up

Out of the 298 women treated for premalignant lesions at MRRH between 2017 and 2020, 227 (76.2%) were lost to follow-up at one year (Figure 1).

Factors associated with loss to follow-up at one year following treatment for premalignant lesions at MRRH

At bivariate analysis (at a significance level of p<0.2), negative HIV serology, receiving thermocoagulation, and failure to attend the review visit at six weeks increased the risk of loss to follow-up at one year, while being more than 45 years old reduced the odds of getting lost to follow-up. The variable “attendance of the review visits at six weeks” was not included in the multivariate analysis because not attending the review visit at six weeks predicted loss to follow-up at one year almost perfectly (RR=2.84, 95% CI: 2.18-3.71, p<0.001). Of the 194 women who did not attend review visits at six weeks, 191 (98.4%) did not attend follow-up at one year. On the other hand, only 36 (34.6%) of the 104 women who attended the review visit at six weeks missed the follow-up visit at one year post-treatment.

At multivariate analysis, only treatment with thermocoagulation (adjusted risk ratio (aRR)=1.21, 95% CI: 1.07-1.36, p=0.003) was associated with loss to follow-up at one year (Table 2).

**Table 2:** Factors associated with loss to follow-up at one year following treatment for premalignant lesions RR: unadjusted/crude risk ratio, CI: confidence interval, aRR: adjusted risk ratio

Characteristic	Loss to follow-up at one year (number (%))	Crude RR	95% CI	p-value	aRR	95% CI	p-value
Age category (years)							
45 and below	217 (77.5)	1	-	-	1	-	-
More than 45	10 (55.6)	0.72	0.47-1.09	0.119	0.74	0.49-1.12	0.151
Area of residence							
Within Mbarara	121 (74.2)	1	-	-	-	-	-
Outside Mbarara	106 (78.5)	1.06	0.93-1.20	0.384	-	-	-
Distance from the hospital							
Less than 10 km	66 (75)	1	-	-	-	-	-
10-30 km	79 (75.2)	1	0.85-1.18	0.97	-	-	-
30-60 km	14 (73.7)	0.98	0.73-1.32	0.906	-	-	-
>60 km	68 (79.1)	1.05	0.90-1.24	0.524	-	-	-
HIV status							
Positive	64 (82.1)	1	-	-	1	-	-
Negative	163 (74.1)	0.9	0.79-1.03	0.124	0.97	0.86-1.10	0.613
Treatment given							
Cryotherapy	149 (71.3)	1	-	-	1	-	-
Thermocoagulation	78 (87.6)	1.23	1.09-1.38	<0.001	1.21	1.07-1.36	0.003
Attendance of review visit at six weeks							
Yes	36 (34.6)	1	-	-	-	-	-
No	191 (98.5)	2.84	2.18-3.71	<0.001	-	-	-

## Discussion

Our study found a very high proportion of loss to follow-up at one year following treatment of women for premalignant cervical lesions at Mbarara Regional Referral Hospital. Receiving thermocoagulation and failing to return for the review visit at six weeks were associated with loss to follow-up at one year.

The proportion of women who did not return for follow-up at one year following treatment for premalignant cervical lesions in this population of Ugandan women is much higher than that found in previous studies [21-23]. It is alarming that more than seven in 10 women who underwent treatment for premalignant cervical lesions did not return for follow-up. The effectiveness of screening in preventing cervical cancer lies in prompt diagnosis and treatment of premalignant cervical lesions [6,12]. Compared to those who screen negative, women who screen positive for premalignant cervical lesions have a 2.5-fold risk of developing cervical cancer, and this is mainly attributable to poor compliance to follow-up following treatment [24]. Without proper follow-up following treatment of premalignant lesions, the intended benefit of reducing the incidence of cervical cancer through screening cannot be achieved.

Missing the review visit at six weeks following treatment for premalignant lesions predicted loss to follow-up at one year almost perfectly. Of the 194 women who did not attend review visits at six weeks, 191 (98.4%) did not attend follow-up at one year. The visit at six weeks following treatment is aimed at identifying and treating post-procedural complications such as infection, persistent vaginal discharge, new-onset vaginal discharge, fever, and bleeding per vaginum. At the same time, the women are counseled further to return for the follow-up visit in one year. Counseling is a time-tested facilitator of adherence to clinical treatment and follow-up [25].

Compared to those who underwent cryotherapy, women who underwent thermocoagulation were more likely not to return for follow-up at one year. Thermocoagulation has a shorter duration of treatment and a much lower rate of side effects such as excessive vaginal discharge and prolonged bleeding compared to cryotherapy [26]. It is possible that the women who underwent thermocoagulation had fewer side effects during recovery and potentially thought that follow-up was not needed. Thermocoagulation, because of its low cost and portability of equipment, has gained preference over cryotherapy, especially in low-income settings [27]. Since most of the patients will be treated with thermocoagulation going forward, measures need to be put in place to enhance patient adherence to follow-up following treatment for premalignant cervical lesions.

Contrary to the findings from a previous study in which long distance was identified as a barrier to adherence to follow-up [28], our study did not find an association between distance to the hospital and compliance with follow-up. Also, there was no difference in compliance with follow-up in regard to age as was previously found in a study done in North Carolina [29]. Compared to those without HIV, people living with HIV have been found to be more compliant with treatments and follow-up in previous studies [30]. Our study however found no relationship between HIV status and loss to follow up.

Strengths and limitations

To the best of our knowledge, this is one of the first studies to document the challenge of loss to follow-up following treatment for premalignant cervical lesions in low-income settings.

However, although our study was able to properly capture the rate of loss to follow-up, we were limited in the exploration of explanatory patient and system factors. This is because our analysis was limited to the information that was obtainable from the clinic registers.

## Conclusions

The proportion of women who did not return for follow-up at one year following treatment for premalignant cervical lesions is very high. Women who do not return for the review visit at six weeks and those who are treated with thermocoagulation are more likely not to return for follow-up at one year. There is a need to implement strategies such as telephone-aided reminders to prompt patients to return for follow-up following treatment for premalignant cervical lesions.

## References

[1] Bhatla N, Aoki D, Sharma DN, Sankaranarayanan R (2021). Cancer of the cervix uteri: 2021 update. Int J Gynaecol Obstet.

[2] Sung H, Ferlay J, Siegel RL, Laversanne M, Soerjomataram I, Jemal A, Bray F (2021). Global cancer statistics 2020: GLOBOCAN estimates of incidence and mortality worldwide for 36 cancers in 185 countries. CA Cancer J Clin.

[3] Arbyn M, Weiderpass E, Bruni L, de Sanjosé S, Saraiya M, Ferlay J, Bray F (2020). Estimates of incidence and mortality of cervical cancer in 2018: a worldwide analysis. Lancet Glob Health.

[4] Yang BH, Bray FI, Parkin DM, Sellors JW, Zhang ZF (2004). Cervical cancer as a priority for prevention in different world regions: an evaluation using years of life lost. Int J Cancer.

[5] IARC Working Group on the Evaluation of Carcinogenic Risks to Humans (2007). Human papillomaviruses. IARC Monogr Eval Carcinog Risks Hum.

[6] Gultekin M, Morice P, Concin N, Querleu D (2020). ESGO contribution to the WHO initiative on elimination of cervical cancer. Int J Gynecol Cancer.

[7] Shiferaw N, Salvador-Davila G, Kassahun K (2016). The single-visit approach as a cervical cancer prevention strategy among women with HIV in Ethiopia: successes and lessons learned. Glob Health Sci Pract.

[8] Nakisige C, Schwartz M, Ndira AO (2017). Cervical cancer screening and treatment in Uganda. Gynecol Oncol Rep.

[9] Chongsuwat T, Ibrahim AO, Evensen AE, Conway JH, Zwick M, Oloya W (2023). Health facility assessments of cervical cancer prevention, early diagnosis, and treatment services in Gulu, Uganda. PLOS Glob Public Health.

[10] Mwaka AD, Garimoi CO, Were EM, Roland M, Wabinga H, Lyratzopoulos G (2016). Social, demographic and healthcare factors associated with stage at diagnosis of cervical cancer: cross-sectional study in a tertiary hospital in Northern Uganda. BMJ Open.

[11] Basu P, Taghavi K, Hu SY, Mogri S, Joshi S (2018). Management of cervical premalignant lesions. Curr Probl Cancer.

[12] World Health Organization, International Agency for Research on Cancer & African Population and Health Research Center (2012). Prevention of cervical cancer through screening using visual inspection with acetic acid (VIA) and treatment with cryotherapy. A demonstration project in six African countries. https://www.who.int/publications/i/item/9789241503860.

[13] World Health Organization (2013). WHO guidelines for screening and treatment of precancerous lesions for cervical cancer prevention. https://apps.who.int/iris/bitstream/handle/10665/94830/978924?sequence=1.

[14] Coker AL, Eggleston KS, Meyer TE, Luchok K, Das IP (2007). What predicts adherence to follow-up recommendations for abnormal Pap tests among older women?. Gynecol Oncol.

[15] Peterson NB, Han J, Freund KM (2003). Inadequate follow-up for abnormal Pap smears in an urban population. J Natl Med Assoc.

[16] Manga S, Kiyang E, DeMarco RF (2019). Barriers and facilitators of follow-up among women with precancerous lesions of the cervix in Cameroon: a qualitative pilot study. Int J Womens Health.

[17] Slavkovsky RC, Bansil P, Sandoval MA (2020). Health outcomes at 1 year after thermal ablation for cervical precancer among human papillomavirus- and visual inspection with acetic acid-positive women in Honduras. JCO Glob Oncol.

[18] (2016). Uganda Ministry of Health: Mbarara Regional Referral Hospital. https://www.health.go.ug/sermon/mbarara-regional-referral-hospital/.

[19] Schmidt CO, Kohlmann T (2008). When to use the odds ratio or the relative risk?. Int J Public Health.

[20] Spiegelman D, Hertzmark E (2005). Easy SAS calculations for risk or prevalence ratios and differences. Am J Epidemiol.

[21] Srisuttayasathien M, Manchana T (2021). Adherence to follow-up in women with cervical intraepithelial neoplasia grade 1. Taiwan J Obstet Gynecol.

[22] Chase DM, Osann K, Sepina N, Wenzel L, Tewari KS (2012). The challenge of follow-up in a low-income colposcopy clinic: characteristics associated with noncompliance in high-risk populations. J Low Genit Tract Dis.

[23] Lendrum T, Alston M, Stickrath E, Rockhill K (2022). Patient adherence to follow-up recommendations following cryotherapy for treatment of high-grade cervical dysplasia. Cureus.

[24] Strander B, Andersson-Ellström A, Milsom I, Sparén P (2007). Long term risk of invasive cancer after treatment for cervical intraepithelial neoplasia grade 3: population based cohort study. BMJ.

[25] M'imunya JM, Kredo T, Volmink J (2012). Patient education and counselling for promoting adherence to treatment for tuberculosis. Cochrane Database Syst Rev.

[26] Chigbu CO, Onwudiwe EN, Onyebuchi AK (2020). Thermo-coagulation versus cryotherapy for treatment of cervical precancers: a prospective analytical study in a low-resource African setting. J Obstet Gynaecol Res.

[27] Castle PE, Murokora D, Perez C, Alvarez M, Quek SC, Campbell C (2017). Treatment of cervical intraepithelial lesions. Int J Gynaecol Obstet.

[28] Chapola J, Lee F, Bula A (2021). Barriers to follow-up after an abnormal cervical cancer screening result and the role of male partners: a qualitative study. BMJ Open.

[29] Michielutte R, Diseker RA, Young LD, May WJ (1985). Noncompliance in screening follow-up among family planning clinic patients with cervical dysplasia. Prev Med.

[30] Kurewa NE, Munjoma MM, Chirenje ZM, Rusakaniko S, Hussain A, Stray-Pedersen B (2007). Compliance and loss to follow up of HIV negative and positive mothers recruited from a PMTCT programme in Zimbabwe. Cent Afr J Med.

